# Sensor Fusion to Estimate the Depth and Width of the Weld Bead in Real Time in GMAW Processes

**DOI:** 10.3390/s18040962

**Published:** 2018-03-23

**Authors:** Guillermo Alvarez Bestard, Renato Coral Sampaio, José A. R. Vargas, Sadek C. Absi Alfaro

**Affiliations:** 1 Postgraduate Program in Mechatronic Systems (PPMEC), Faculty of Technology, University of Brasilia, Campus Darcy Ribeiro, Brasilia 70910-900, Brazil; 2 Department of Automatic Control, Institute of Cybernetics, Mathematics and Physics, Havana 10400, Cuba; 3 Software Engineering Group, Faculty of Gama, University of Brasilia, Brasilia 72444-240, Brazil; renatocoral@unb.br; 4 Department of Electrical Engineering, Faculty of Technology, University of Brasilia, Brasilia 70910-900, Brazil; vargas@ene.unb.br; 5 Department of Mechanical and Mechatronic Engineering, Faculty of Technology, University of Brasilia, Brasilia 70910-900, Brazil; sadek@unb.br

**Keywords:** arc welding process, bead depth estimation, bead width estimation, FPGA, GMAW, neural network, sensor fusion

## Abstract

The arc welding process is widely used in industry but its automatic control is limited by the difficulty in measuring the weld bead geometry and closing the control loop on the arc, which has adverse environmental conditions. To address this problem, this work proposes a system to capture the welding variables and send stimuli to the Gas Metal Arc Welding (GMAW) conventional process with a constant voltage power source, which allows weld bead geometry estimation with an open-loop control. Dynamic models of depth and width estimators of the weld bead are implemented based on the fusion of thermographic data, welding current and welding voltage in a multilayer perceptron neural network. The estimators were trained and validated off-line with data from a novel algorithm developed to extract the features of the infrared image, a laser profilometer was implemented to measure the bead dimensions and an image processing algorithm that measures depth by making a longitudinal cut in the weld bead. These estimators are optimized for embedded devices and real-time processing and were implemented on a Field-Programmable Gate Array (FPGA) device. Experiments to collect data, train and validate the estimators are presented and discussed. The results show that the proposed method is useful in industrial and research environments.

## 1. Introduction

Arc welding is extensively used in industrial applications. Among all welding methods, the Gas Metal Arc Welding (GMAW) process is used in automotive, aeronautic and naval industries. In the energy industries, it is applied in the manufacturing of thermoelectric, hydroelectric, nuclear and wind farms. It is also used in the production of tools and instruments for the scientific, industrial, domestic and medical sectors. Other applications include the construction of civil and military structures and more specifically in the manufacturing of pipes to fluids transportation such as gas, oil and water. Among the GMAW strengths is its versatile welding process that yields high-quality weld beads.

In the process, the geometry of the weld bead is critical and commonly used for quality validation, but an online measurement is challenging due to the extreme environmental conditions imposed on the welding arc [1]. These characteristics limit the process automation since the control loop cannot be closed with classical instrumentation techniques and requires non-contact measuring techniques, such as video or thermographic cameras, microphones, spectrometers, X-rays, ultrasound, among others.

The weld bead geometry includes width, reinforcement and depth of penetration [2]. These parameters are governed by several process factors such as wire feed speed, welding speed, welding current, welding voltage and contact tip to work distance.

The sensing and analysis techniques used in the welding process is presented in [3]. Among all studied references, only 15% use dynamic process models due to the difficulty in obtaining a continuous data set of weld bead depth. In several control systems, the static model is not acceptable for satisfactory performance. In addition, it should be noted that the vision, infrared thermography and sensor fusion techniques combined with neural network, image processing and statistical analysis, are the most commonly used in more recent works.

Vision sensing techniques are a good solution to measure the weld bead width and reinforcement but are not suitable for measuring the weld bead depth. One common method uses a laser beam that draws one or more lines or dots on the weld bead while a camera captures the image created by them. A band-pass optical filter is used to isolate the lines emitted by the laser. This technique uses special lenses, image processing algorithms and triangulation techniques. The final result is a two-dimensional or three-dimensional profile of the weld bead or weld pool with geometric information [4,5,6,7,8]. This system is referred to as profilometer, profile sensor or laser scanner.

Infrared thermography provides a non-contact measurement of the molten weld pool infrared emission that is strongly correlated with the temperature. The infrared sensing of arc welding processes has been extensively investigated and results prove that the information provided by this measurement technique can help determinate the weld bead depth and width [9,10,11,12,13,14]. Several patents have been recognized in the United States of America to detect and control total penetration using infrared information [15,16,17].

Sensor fusion techniques are used to describe the static and dynamic behaviour of process variables [18,19,20,21]. These techniques are increasingly being used to estimate magnitudes that are impossible to measure directly. Sensor fusion is based on several other areas of knowledge, such as statistics and artificial intelligence, but it finds new applications in this field.

By combining different sensing technologies to take advantage of each one’s strength, it is possible to obtain better sensing results. As an example, the weld pool vision and infrared information can be combined to obtain the weld pool dimensions with more accuracy, or the combination of the weld bead width (from a vision system) and the back bead thermographic information (from a pyrometer) to estimate the bead penetration [22,23]. Nowadays, because of these and other advantages, sensor fusion systems were studied to obtain an effective weld pool and weld bead estimations [24,25,26,27,28,29,30,31].

Despite the advantages and the success of these methods in a scientific environment, their application in an industrial environment is low or null. Related works do not show the application of embedded devices to bead geometry control in the arc welding process and an overview shows that the implementation of welding geometry control systems does not find support in the embedded devices area. Other welding related works such as weld pool volume control [32], image processing [33], wire feed control [34], defect detection [35,36,37] and arc signal monitoring [38,39], were found. This indicates that the implementation of sensor fusion algorithms in embedded devices to estimate the geometry of the weld bead can become a new field of application for these technologies.

This paper presents a system developed to stimulate a GMAW conventional process and collect values of the arc variables, infrared thermography and weld bead geometry. The GMAW process uses a constant voltage power source. The system allows real-time open-loop control of the process and stores experimental data on a computer. Image processing algorithms were developed to extract features from the thermographic data, measure the external weld bead dimensions and depth by making a longitudinal cut in the weld bead. This information is used to train a multilayer perceptron neural network, which is based on a sensor fusion algorithm to estimate the depth and width of the weld bead that can be used in real-time. The inputs of the neural network add previous measurements and estimated values to capture the process dynamics. The estimators are implemented, simulated and tested in a Field-Programmable Gate Array (FPGA) embedded system. The actual application area is automatic control, arc welding and sensor fusion research.

In this context, we can cite three main contributions of this work. First is a novel modelling method which improves the model response by adding dynamic process information rather than relying on static models used in most works found in the literature. This is achieved by the image processing algorithm to the macrographic analysis developed in this work that collects a continuous data set of weld bead depth, necessary to the dynamic model.

The second main contribution is a novel method, also used in modelling, that obtains the thermographic features and supplies information about the amount and spatial distribution of the energy in the workpiece. This method does not use the second derivate to thermographic width calculation, reducing the mistakes when multiple inflexion points are found. Also, a real thermographic curve is used to volume calculations instead of the ideal Gaussian curve. This approach improves model accuracy and simplifies the calculations, reducing it just to sum operations. Other works use the Gaussian curve to thermographic profile representation and need more complex equations.

The third main contribution is the floating-point FPGA implementation of the artificial neural network (ANN) equations that supplant the need for normalization steps by incorporating them in the weights of the ANN. The tests of the estimators in the FPGA device show a low response time and are suitable for use in real-time.

## 2. Materials and Methods

In this work, a data acquisition and open-loop control system was developed. The next sections describe this system and the analysis methods to measure the relevant data and estimate the depth and width of the weld bead.

### 2.1. Data Acquisition and Open-Loop Control System for GMAW Welding Process

This experimental system was developed to collect values of selected variables and stimulate the GMAW process, allowing an open-loop control and data storage in a computer.

The variables measured by the system are welding voltage, welding current, infrared thermography and welding torch (or workpiece) position. The system sends several set points to the welding power source such as the set point of welding voltage and the set point of the wire feed speed and controls the welding speed. Despite this, the control is in open loop because the system does not use the measurements to change the welding parameters and follows a sequence previously defined by the system operator.

**Figure 1:**
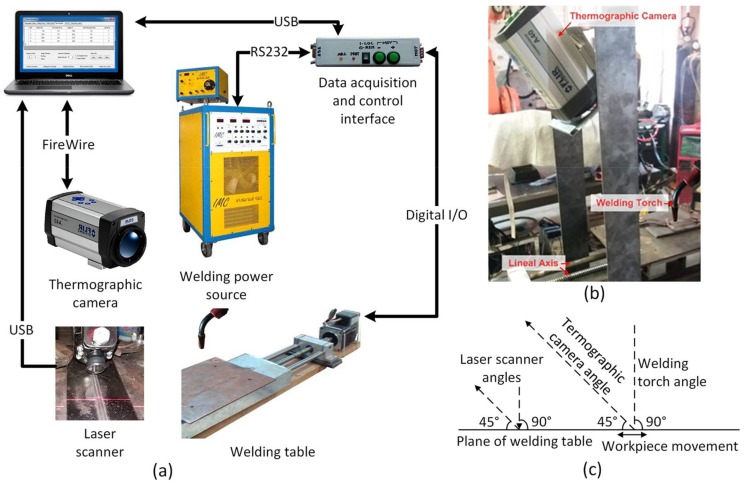
Data acquisition and open loop control system: (**a**) Main components; (**b**) Flat welding table and instruments support; (**c**) Schematic side view.

The data acquisition and open-loop control system has six main components: the welding power source, the welding table, the thermographic camera, the laser scanner, the data acquisition and control interface and the computer where data acquisition and stimulus sequence design software are executed [40]. The components, the data flow and a schematic side view are shown in Figure 1.

The *welding power source* used is the Inversal 450 [41]. The communication algorithm for remote control of the welding power source and data acquisition was developed, based on the RS232 protocol [42]. A state machine, implemented in the control interface, defines the operation parameters and obtains the arc variables measurements (welding voltage and welding current) and status of the welding power source. The operation sequence for controlling the welding power source and the data acquisition software was also developed [40]. A state machine is used to continuously repeat this sequence in the main loop of the interface.

The electro-mechanical system of the *flat welding table* is composed of a linear axis with 5 mm of linear movement by revolution and a stepper motor of 1.8 grades by step. The system is used to move the piece, keeping the welding torch fixed. It was developed by students of the Automation and Control Group (GRACO) of UnB. It has a stepper motor control circuit [43] with signals for modifying the stepper time (speed) and the direction. Other signals show the status of the system and protect against overload [44]. The structure supports 15 kg of load and up to 15 mm/s of speed. The support structure for the thermographic camera was developed in this work and is shown in Figure 1b.

**Figure 2:**
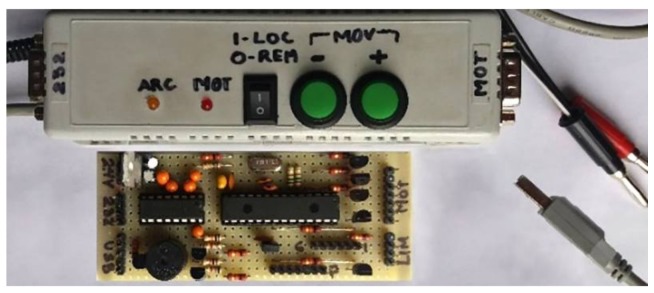
Data acquisition and control interface.

The *data acquisition and control interface* was developed to synchronize the movement of the piece with the parameters of the welding power source operation and to obtain the arc variables measurements in real-time [40]. Figure 2 shows the simplicity and small size of the interface (since its greater complexity is in the firmware) as well as the low cost of its components.

A microcontroller PIC18F2550 from Microchip Technology [45] (Arizona, USA, imported to Brazil by Ichip Tecnologia Ltda—ME) controls the operation. It first receives from the computer the start and end points of the weld, and the sequence of values to be sent to the welding source in each position of the piece. Then, after starting, the interface has an autonomous and independent operation and sends the measurements obtained in each position in real-time to the computer. The acquisition program (synchronized with the interface) obtains the thermographic data regardless of the clock or priorities of the operating system. The USB communication frames between the interface and the computer were created, based on ASCII characters. This allows the control of the welding power source, the welding table, the interface and the operation sequence of the process.

The *thermographic camera* ThermoVision A40 [46] from FLIR Systems (Oregón USA, imported to Brazil by FLIR Systems Brazil) is used to obtain the temperature values of the weld molten puddle. It employs a semiconductor sensor of focal plane array uncooled microbolometer technology; has a temperature range between −40 °C and 2000 °C; a spectral range between 7.5 and 13 μm; a sampling frequency of 120 Hz (120 frames per second) and a maximum resolution of 16-bit monochrome and 8-bit colour. The infrared data is obtained from a Firewire interface [47] of the computer in a matrix format, with the temperature of each pixel of the image. The camera is fixed at 50 mm from the molten weld pool at a 45° angle from the horizontal plane of the welding table (see Figure 1b,c).

The emissivity of the molten pool is not constant, so the temperature measurements using this method should be considered approximate. The accurate measuring of the absolute values of temperatures is not considered significant for the purposes of this work.

**Figure 3:**
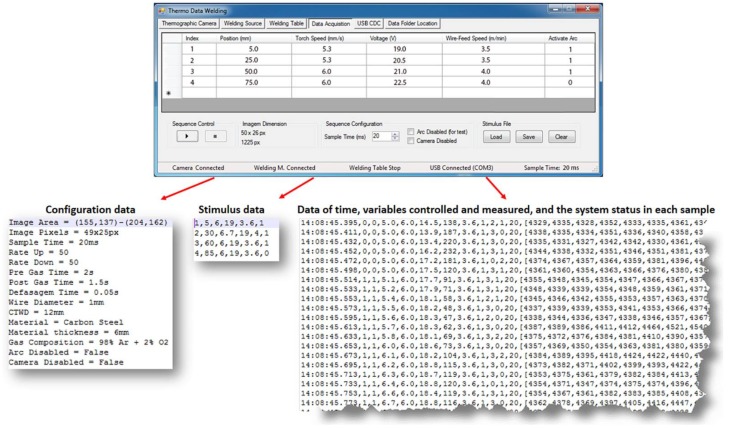
Data acquisition and stimulus sequence design form and data files.

The *data acquisition and stimulus sequence design software* developed using the libraries provided by the thermographic camera manufacturer [48]. The software Thermo Data Welding (TDW) developed in this work collects and stores the data of the welding process and operation of the whole system in text files. The data are collected using the communication links with the data acquisition interface and the thermographic camera (see Figure 1a). Figure 3 shows the primary form of TDW.

The TDW is divided in several modules oriented to specific functions such as: thermographic camera configuration and verification, welding power source configuration (defines inductance, pre-gas and post-gas times, gap wire-arc time, wire diameter, contact tip to work distance, type and thickness of the material, composition and flow rate of the shielding gas), adjustment of the initial position of the piece in the welding table, creation the welding sequence (defines start and end positions, stimulus that will be sent to the welding power source and the sampling period). Three files are created in each experiment (see Figure 3 bottom) that store the system configuration, the stimulus sequence and the measurements collected.

### 2.2. Infrared Image Features Extraction

**Figure 4:**
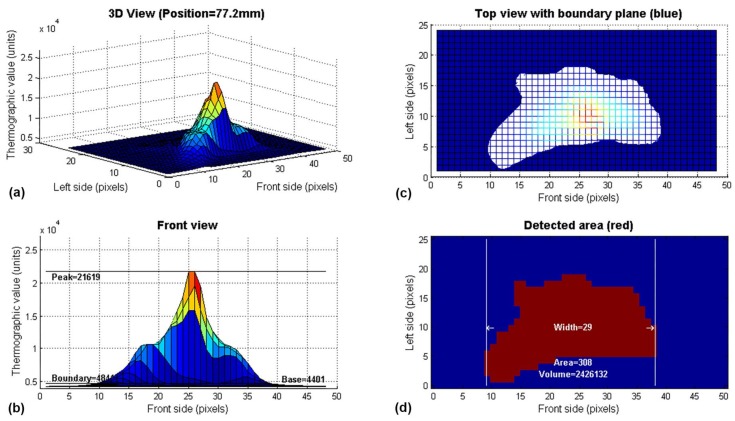
Graphical representation of the infrared image features extracted from a sample: (**a**) 3D curve of the thermographic values; (**b**) Front view of the curve with peak, boundary and base values; (**c**) Intersection between the boundary plane and the 3D curve; (**d**) Area, volume and width detected.

The thermographic matrix (supplied by the thermographic camera) is processed with an algorithm developed to obtain the next characteristics in each sampling time: thermographic peak (*T_p_*), base plane (*T_b_*), thermographic curve width (*T_w_*), thermographic area (*T_a_*) and thermographic volume (*T_v_*). The thermographic image is taken on the weld pool area as a physical reference, but the welding arc and the electrode are included. The developed algorithm tries to obtain information about the amount and spatial distribution of the energy supplied to the workpiece to estimate the depth and width of the weld bead. An example of the algorithm output for one sample is shown in Figure 4.

The data processing algorithm applies a moving average filter of 3 × 3 pixels to the infrared matrix. The thermographic peak or maximum value of the matrix is calculated. The base plane is calculated as the average of 10% of the values in the left and right edges (left and right side, see Figure 4b). The boundary plane is 10% above the base plane.

The sum of active pixels in the intersection plane between the thermographic surface and the boundary plane is the area. The sum of the thermographic values within the intersection plane is the thermographic volume (see Figure 4d).

In the image shown in Figure 4b, it is possible to observe several inflexion points in pixels 17 and 19. This characteristic is observed in several images of the thermographic profile. It makes inexact the use of the second derivative analysis to determine the width of the thermographic profile. In this work, to prevent this problem, the thermographic width is the maximum count of active pixels in the front axis (front side) of the intersection plane, as shown in Figure 4d.

This algorithm was developed using Matlab scripts but is very simple and can be implemented in an FPGA or microcontroller device.

### 2.3. Laser Scanner and Macrographic Analysis to Obtain the Geometric Profile of the Weld Bead

The laser scanner and the macrographic analysis algorithm are a hardware and software development to obtain the profile of the weld bead after the completion of the welding process. The laser scanner gets the three-dimensional external geometry and the macrographic analysis algorithm obtains the weld bead depth profile.

**Figure 5:**
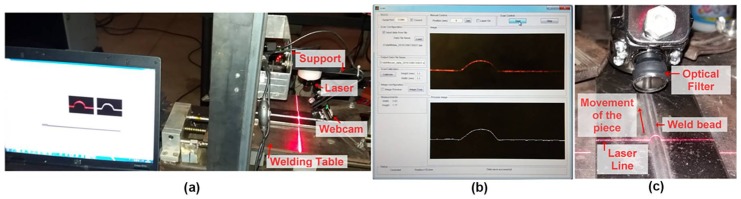
Low-cost laser scanner: (**a**) Main components; (**b**) Software for data acquisition and image processing; (**c**) Details of the optical components and the laser line.

The *laser scanner* (or profilometer) operation is based on a triangulation technique and image processing algorithms developed specifically for this system. It draws a line on the weld bead with a laser and a low-resolution camera (webcam of 600 × 400 pixels, 0.24 MP) with USB communication captures the image. The camera provides only the laser line image because an optical filter, adjusted to the laser emission frequency of the laser, blocks other light sources. Figure 5 show this process.

To operate the scanner, after the welding finishes the workpiece remains fixed on the welding table. Then, the control interface moves into the scanning position. A software developed using Matlab/GUI acquires the image from the camera and applies the image processing algorithm. The algorithm filters the image and completes the missing data, then rotates the image to find the baseline corresponding to the base metal surface and calculates the reinforcement and width of the bead. Figure 5b shows the image obtained by the camera for the current position in red and the calculated profile in white.

The system saves pixel resolution, position information, reinforcement, width and profile of the weld bead for each position in a text file. These data are useful for three-dimensional reconstruction and statistical analysis of the weld bead geometry. The system can scan a weld bead up to approximately 20 mm wide and 10 mm high. The calibration is done with an automatic procedure using pieces with known dimensions. A resolution of 0.035 mm of width and 0.05 mm of height has been verified with an error of less than 5% of the full scale. This is an acceptable low-cost solution for research purposes despite its slow performance and low image resolution.

**Figure 6:**
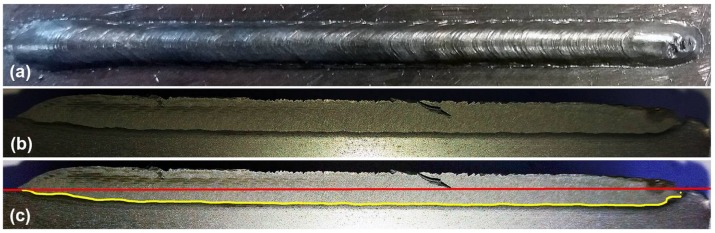
Macrographic analysis applied in a weld bead: (**a**) Top view of the weld bead; (**b**) Original image of the lateral view of the cut and polished piece; (**c**) Image processed by the algorithm that shows the depth profile of the bead (yellow line) and the surface of the workpiece (red line).

The *macrographic analysis algorithm* was developed using Matlab scripts and needs an image of the welding workpiece. The workpiece is cut in a longitudinal direction, that is, in the direction of the torch movement (see Figure 6a,b). If a misalignment of the cut tool with the weld bead is detected, it may be necessary to perform more than one cut-analysis operation. In these cases, the maximum value of each position is used.

The cut piece will be polished and etched using Nital solution (usually 2.5%) to clearly show the weld bead penetration, as can be seen in Figure 6b. An image processing algorithm analyses the image of this side of the piece, then corrects misalignment and filters the image for the border detection procedure. The base-line (shown in red in Figure 6c) is detected. The thickness and length of the piece are known and are used to calculate the scale coefficients. The weld bead penetration limit zone is detected (shown in yellow in Figure 6c) and the difference between it and the base-line returns as the weld bead depth profile. The profile data is filtered, and the missing values are filled with a linear interpolation method.

In the traditional transversal cut, is not possible to obtain enough information to make a dynamic model because of the difficulty to obtain a continuous data set of penetration. This macrographic analysis algorithm allows us to obtain a continuous data set of penetration to a dynamic model.

### 2.4. Sensor Fusion to Estimate the Depth and Width of the Weld Bead

A cooperative, data-in/data-out and centralized sensor fusion [18] algorithm was developed based on a Multilayer Perceptron (MLP) artificial neural network to estimate two characteristics of the weld bead geometry. The inputs of the neural network add previous measurements and estimates values to capture the process dynamics.

**Figure 7:**
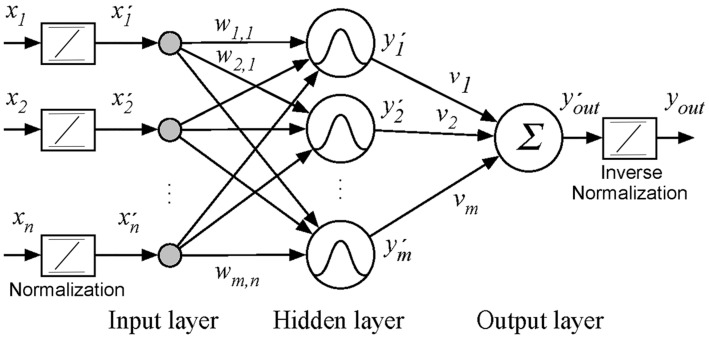
Structure of multilayer perceptron neural network.

The MLP is composed of an input layer, one or more hidden layers and an output layer. This work uses only one hidden layer and a single output as shown in Figure 7.

For a better training performance, all inputs have to be normalized in the interval [−1, 1] by Equation (1). In this equation *x_i_ =* [*x*_1_, *x*_2_, … *x_n_*] are the *n* inputs and *x’_i_ =* [*x’*_1_, *x’*_2_, … *x’_n_*] represent the normalized values.

(1) $$x{’}_{i}=2\left(\frac{x_{i}-\min\left(x_{i}\right)}{\max\left(x_{i}\right)-\min\left(x_{i}\right)}\right)-1$$

In mathematical terms, Equation (2) represents the input of a neuron in the hidden layer where *w_i,j_* is the weight of neuron *j* associated with the input *i*, and *b_j_* is the bias of neuron *j*. Equation (3) expresses the output of the hidden layer where *f* is the *tansig* activation function presented in Equation (4).

(2) $$s_{1,j}=\left(\overset{n}{\underset{i=1}{\sum }}w_{i,j}x{’}_{i}\right)+b_{j}$$

(3) $$y{’}_{j}=\;f\left(s_{1,j}\right)=f\left[\left(\overset{n}{\underset{i=1}{\sum }}w_{i,j}x{’}_{i}\right)+b_{j}\right]$$

(4) $$f=\;tansig\left(s\right)=\frac{2}{1+e^{-2s}}-1$$

Equation (5) represents the output layer of the neural network where *y’_j_* and *v_j_* are respectively the output and weight associated with neuron *j* of the hidden layer, and *b_out_* is the output bias.

(5) $$y{’}_{out}=\left(\overset{m}{\underset{j=1}{\sum }}v_{j}y{’}_{j}\right)+b_{out}$$

Finally, the output *y’_out_* has to be restored to the original interval (inverse normalization), which is done through Equation (6).

(6) $$y_{out}=\left(\frac{y{’}_{out}+1}{2}\right)\left[\max\left(y_{out}\right)-\min\left(y_{out}\right)\right]+\min\left(y_{out}\right)$$

**Figure 8:**
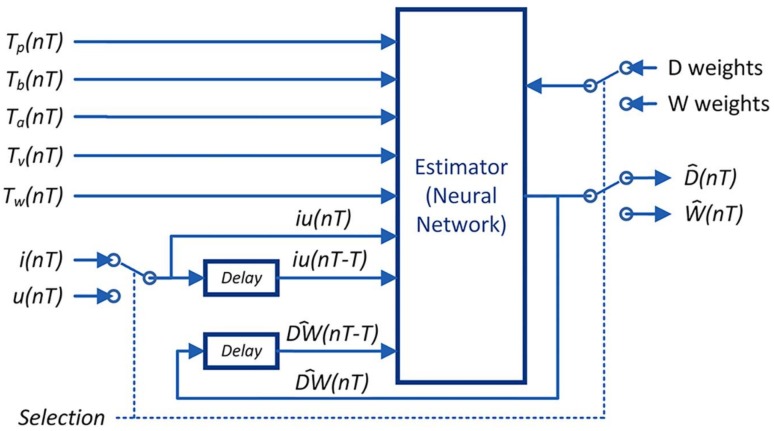
Estimator of depth and width of the weld bead that uses the thermographic features with welding current or voltage in cooperative mode.

The same MLP structure is used to estimate both the weld bead depth ($\hat{D}$) and the weld bead width ($\hat{W}$) by changing the input parameters of the welding current (*i*) to the welding voltage (*u*). A selection signal is used to change the parameters (weights), the inputs and the output of the network. A controller evaluates the two algorithms in sequence, updating the weights of the neural network for each case. Figure 8 shows the block diagram of the estimator.

The artificial neural network has eight neurons in the input layer, twelve in the hidden layers and one in the output layer. The size of the hidden layer was selected by balancing the computational cost (neuron quantity) and the estimation error by testing different configurations.

The input variables are the thermographic peak (*T_p_*), the thermographic base (*T_b_*), the thermographic area (*T_a_*), the thermographic volume (*T_v_*), the thermographic width (*T_w_*), the measurements of welding current or voltage (*iu*) in the actual (*nT*) and previous sample (*nT-T*) and the previous estimated value [$\hat{DW}\left(nT-T\right)$]. The symbol *T* is the sample time and n is the sample number. The activate function is the hyperbolic tangent sigmoid transfer function [49]. The network training should be done with experimental measurements of the parameters of input and output, and by using the backpropagation algorithm.

The *Selection* signal allows switching between the estimation of the weld bead depth or the weld bead width. To estimate the weld bead depth, this algorithm uses the features of the infrared image and measurements of the welding current as indicators of the energy delivered (welding energy) and the last value of the weld bead depth. To estimate the weld bead width, the infrared features, the welding voltage and the last value of the weld bead width are used.

For the training and validation of the neural network, the input values of the thermographic and arc variables are obtained from the data acquisition system (see Section 2.1) and infrared feature extraction algorithm (see Section 2.2). The real values of the weld bead depth are obtained from the macrographic analysis. The real values of the weld bead width are obtained from the laser scanner (see Section 2.3).

These dynamic estimator’s models are valid only for the current process conditions and must be updated if these change, such as electrode diameter and material, base material, gas type and flow rate, welding current or welding voltage range, among others.

Several programs were developed for data processing and data plotting. All the curves, the statistical analysis, the image processing and the neural network training and testing were made using Matlab scripts.

We must emphasize the importance of simplifying the estimating algorithm due to real-time requirements. The use of the proposed parameters and not the complete infrared image can help reduce the resources used and increase the speed of the estimation process. The same structure for the estimation of two parameters in sequential mode minimizes the hardware requirements but increases the processing time. If less processing time is required and the hardware resource is not restricted, two equal neural networks can be used. The implementation and synthesis of the neural network can be done with the methodology and tools proposed in [50,51]. Ayala et al., show a floating-point implementation in hardware for a neural network architecture that guarantees low latency.

### 2.5. FPGA Implementation of Weld Bead Depth and Width Estimators

**Figure 9:**
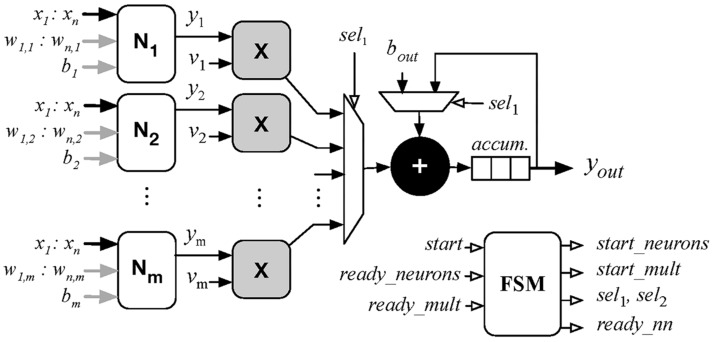
Blocks diagram of neural network implementation in Field-Programmable Gate Array (FPGA) device using a Finite State Machine (FSM).

The MLP implementation in hardware is based on the model presented in Section 2.4. The overall architecture consists of multiple neurons in parallel, which are synchronized and controlled by a Finite State Machine (FSM) as shown in Figure 9. All operations are carried out using customized variable width floating-point arithmetic and trigonometric libraries based on the IEEE 754 standard. These units are described in [52,53] and provide much better precision and a larger dynamic range suited for small and large real numbers compared to fixed-point or simple integer arithmetic.

The top-level neural network architecture begins its operation with the activation of the *start* signal, at this point, the FSM sends a *start_neuron* command to each neuron which receives the network inputs and its respective weights (*a*) and bias (*b*) The neuron then outputs its value (*y*) and activates a *ready_neuron* signal. The FSM then activates a *start_mult* signal which begins multiplying each neuron output by its respective weight (*v*). After receiving the *ready_mult* signal, the FSM executes its final states which sums all neuron outputs multiplied by their weights with the output bias (*b_out_*). Finally, the *ready_nn* signal is set and the network output (*y_out_*) becomes available.

**Figure 10:**
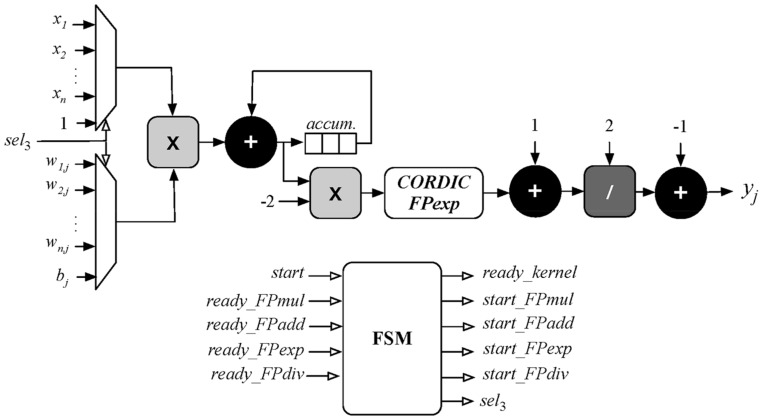
Block diagram of neuron implementation in FPGA device.

The neuron module has a similar architecture and is depicted in Figure 10. Again, an FSM receives a *start* signal and begins the first stage of multiplying each input with its respective weight and accumulating the results. To simplify the architecture, the bias value (*b_j_*) is inserted in the same pipeline and multiplied by 1. After that, the resulting value enters the activation function as described in Equation (4). The exponential operation uses a CORDIC module described in [53] while the rest of the arithmetic operators use the same floating-point units described in [52].

To optimize FPGA resource usage, all sequential operations inside the neuron use shared floating-point units which reduces them to a total of two adders, one multiplier and one divider. Likewise, the multipliers used in the top-level architecture are also shared with the multipliers inside each neuron avoiding resource duplication.

The final step to the MLP hardware implementation was to address the input and output normalization steps described in Equations (1) and (6). To avoid adding more logic to the MLP architecture in hardware, a transform operation was applied to each weight and bias. To calculate the transforms, we began by substituting Equation (1) into Equation (2) and obtaining the Equation (7).

(7) $$s_{1,j}=\left(\overset{n}{\underset{i=1}{\sum }}w_{i,j}\left(2\left(\frac{x_{i}-\min\left(x_{i}\right)}{\max\left(x_{i}\right)-\min\left(x_{i}\right)}\right)-1\right)\right)+b_{j}$$

(8) $$s_{1,j}=\left(\overset{n}{\underset{i=1}{\sum }}w_{i,j}\left(\frac{2x_{i}}{\max\left(x_{i}\right)-\min\left(x_{i}\right)}-\frac{2\min\left(x_{i}\right)}{\max\left(x_{i}\right)-\min\left(x_{i}\right)}-1\right)\right)+b_{j}$$

(9) $$s_{1,j}=\;\left(\overset{n}{\underset{i=1}{\sum }}\frac{2x_{i}w_{i,j}}{\max\left(x_{i}\right)-\min\left(x_{i}\right)}-\frac{2\min\left(x_{i}\right)w_{i,j}}{\max\left(x_{i}\right)-\min\left(x_{i}\right)}-w_{i,j}\right)+b_{j}$$

(10) $$s_{1,j}=\overset{n}{\underset{i=1}{\sum }}x_{i}\frac{2w_{i,j}}{\max\left(x_{i}\right)-\min\left(x_{i}\right)}-\overset{n}{\underset{i=1}{\sum }}\left(\frac{2\min\left(x_{i}\right)w_{i,j}}{\max\left(x_{i}\right)-\min\left(x_{i}\right)}-w_{i,j}\right)+b_{j}$$

From Equation (10) it is clear that the neuron weights and bias can be recalculated to include the normalization as expressed in Equations (11) and (12).

(11) $$w\_norm_{i,j}=\frac{2w_{i,j}}{\max\left(x_{i}\right)-\min\left(x_{i}\right)}$$

(12) $$bias\_norm_{j}=-\overset{n}{\underset{i=1}{\sum }}\left(\frac{2\min\left(x_{i}\right)w_{i,j}}{\max\left(x_{i}\right)-\min\left(x_{i}\right)}-w_{i,j}\right)+b_{j}$$

An equivalent transform was calculated for the output inverse normalization where Equation (5) is substituted into Equation (6).

(13) $$y_{out}=\left(\frac{\left(\sum_{j=1}^{m}v_{j}y{’}_{j}\right)+b_{out}+1}{2}\right)\left(\max\left(y_{out}\right)-\min\left(y_{out}\right)\right)+\min\left(y_{out}\right)$$

(14) $$y_{out}=\frac{\left(\sum_{j=1}^{m}v_{j}y{’}_{j}\left(\max\left(y_{out}\right)-\min\left(y_{out}\right)\right)\right)}{2}+\frac{\left(b_{out}+1\right)\left(\max\left(y_{out}\right)-\min\left(y_{out}\right)\right)}{2}+\min\left(y_{out}\right)$$

Once again, from Equation (14) it is possible to isolate the expressions that transform the weights and bias used to compute the inverse normalization.

(15) $$v\_nomr_{j}=\frac{\left(\max\left(y_{out}\right)-\min\left(y_{out}\right)\right)}{2}v_{j}$$

(16) $$b\_norm_{out}=\frac{\left(b_{out}+1\right)\left(\max\left(y_{out}\right)-\min\left(y_{out}\right)\right)}{2}+\min\left(y_{out}\right)$$

With the use of these transforms, there is no need to add any logic to the MLP implemented in hardware.

## 3. Results and Discussion

Two welding experiments were completed for depth and width of the weld bead modelling and validation. In the following sections, we show the details of the experiments, the measurements obtained, modelling and validation of geometry estimators and an FPGA simulation of the estimators.

### 3.1. Experimental Details

The welding experiments were made using carbon steel pieces with dimensions of 85 × 50 × 6 mm on which welds were laid adopting the bead-on-plate technique (welding on top). The surfaces of the pieces were cleaned to eliminate any dirt and oxides. The piece was welded in a horizontal position, with the torch at 90° from the horizontal plane and 12 mm of contact tip to work distance (CTWD). The shielding gas was composed of 98% Argon and 2% Oxygen, with 15 psi of pressure.

The selection of the welding electrode wire was based mainly upon matching the mechanical properties and physical characteristics of the base metal, weld size and existing electrode inventory. Steel wires with a diameter of 1 mm (Product code CAJMERS6100S015W00 of Merit^®^S-6 Lincoln Electric from São Paulo, Brazil) were used.

The measurements were obtained using the data acquisition and control system described in Section 2.1. The weld bead profile was obtained using the laser scanner and macrographic analysis described in Section 2.3. The welding power source was configured (through the data acquisition and control system) with a rate up and a rate down of 50, 2 s of pre-gas time, 1.5 s of post-gas time and 0.05 s of gap time. The sampling time was 20 ms. The dimensions of the thermographic image were 49 × 25 pixels.

#### 3.1.1. Experiment 1: Process Response to the Step in the Welding Speed and Wire Feed Speed

**Figure 11:**
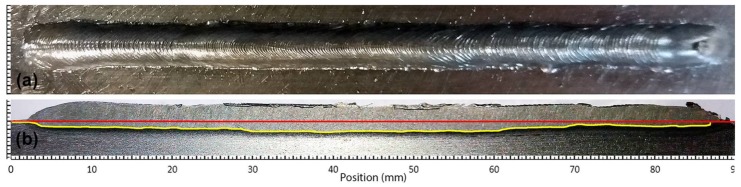
Weld bead obtained in the experiment with variations in the welding speed and wire feed speed: (**a**) Top view of the weld bead; (**b**) Weld bead depth profile (yellow line) and the surface of the workpiece (red line) obtained by the macrographic analyses algorithm.

**Figure 12:**
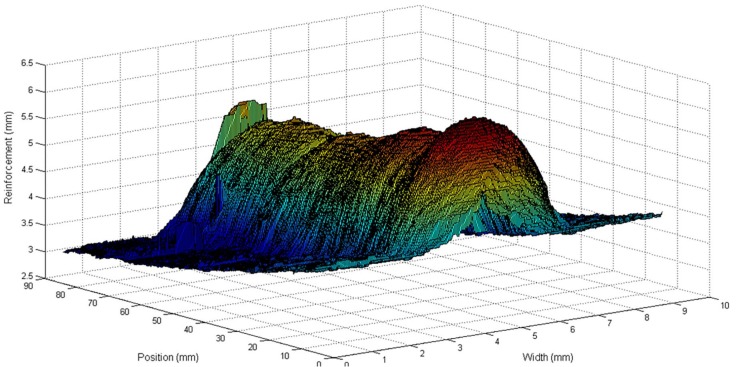
Three-dimensional reconstruction of the weld bead obtained in the experiment with variations in the welding speed and wire feed speed.

This experiment was performed to investigate the behaviour of the process with welding speed and wire feed speed variations. These variations were made by keeping the deposited volume by area unit constant by keeping the ratio between the welding speed and wire feed speed constant. The welding voltage was also constant. The step input sequence used in this experiment is shown in Table 1. The weld bead obtained is shown in Figure 11 and the three-dimensional weld bead profile is shown in Figure 12.

**Figure 13:**
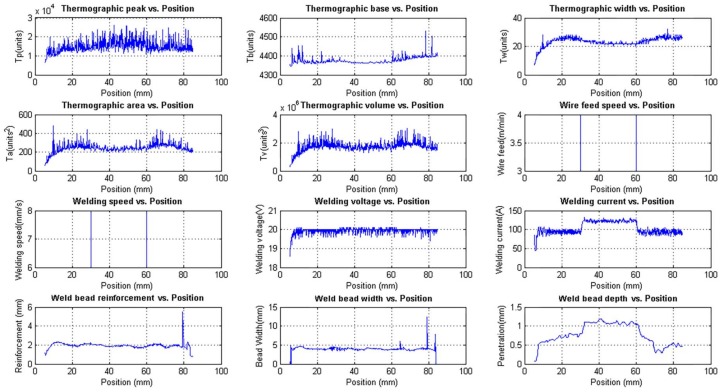
Unfiltered experimental measurements obtained with the data acquisition and control system varying the welding speed and wire feed speed.

**Figure 14:**
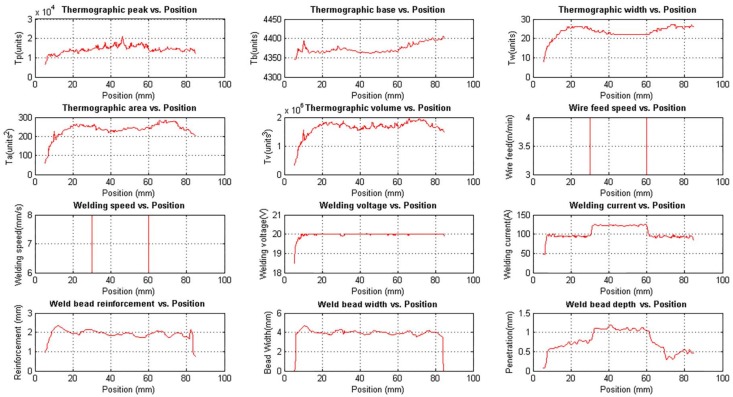
Filtered experimental measurements obtained with the data acquisition and control system varying the welding speed and wire feed speed.

Figure 13 shows the unfiltered data and Figure 14 shows the filtered data. The data used for modelling is unfiltered but can be filtered in the modelling algorithm if necessary. In these figures, it is possible to notice that, although the wire feed speed has changed, the bead width and the bead height remain stable because of the constant relationship between the wire feed speed and the welding speed and the constant value of the welding voltage.

The correlation between the wire feed speed and the welding current is observed in the figures. When the wire feed speed is increased the welding current increases too. The relationship between the weld bead depth and the welding current is clear although the depth reaches a small value. This is observed in Figure 14.

The autocorrelation and cross-correlation analysis show high values, indicating an adequate sampling time and a delay of 11 sampling times between the weld bead depth and the stimulus.

#### 3.1.2. Experiment 2: Process Response to the Step in the Welding Voltage

**Figure 15:**
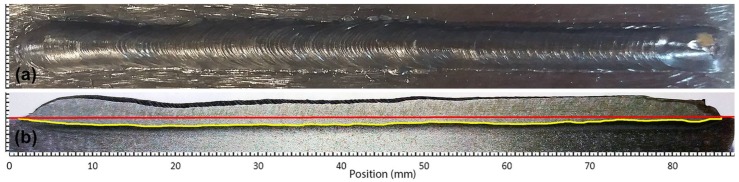
Weld bead obtained in the experiment with variations in the welding voltage: (**a**) Top view of the weld bead; (**b**) Weld bead depth profile (yellow line) and the surface of the workpiece (red line) obtained by the macrographic analyses algorithm.

**Figure 16:**
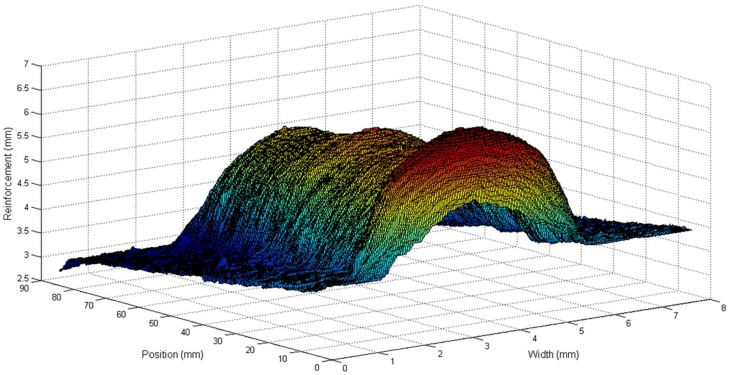
Three-dimensional reconstruction of the weld bead obtained in the experiment with variations in the welding voltage.

This experiment was performed to investigate the behaviour of the process with welding voltage variations. The wire feed speed and the welding speed were constant. The step input sequence used in this experiment is shown in Table 2. The weld bead obtained is shown in Figure 15 and the three-dimensional weld bead profile is shown in Figure 16.

**Figure 17:**
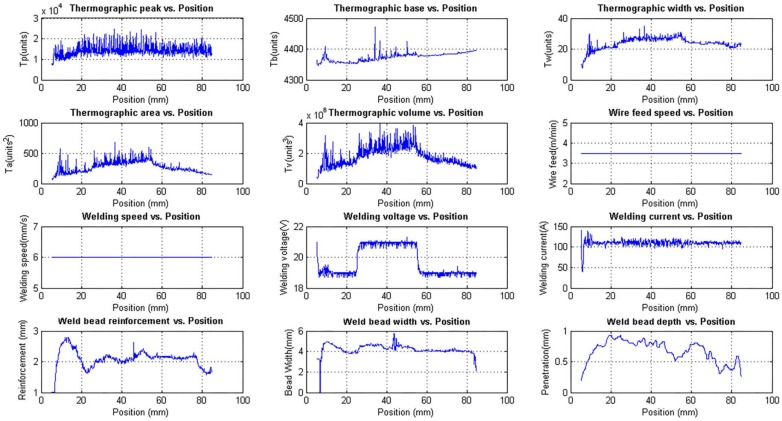
Unfiltered experimental measurements obtained with the data acquisition and control system varying the welding voltage.

**Figure 18:**
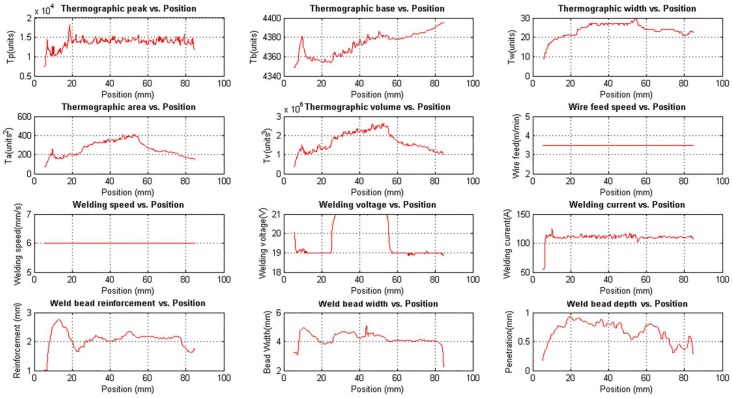
Filtered experimental measurements obtained with the data acquisition and control system varying the welding voltage.

Figure 17 shows the unfiltered data and while Figure 18 shows the filtered data. The correlation between the welding voltage and the thermographic parameters is observed in the figures. When the voltage is increased, the thermographic width increases too. In consequence, the area and volume are increased.

All thermographic parameters were calculated from the base value, except the thermographic peak. When the base value changes, the width, area and volume are inversely affected. In this experiment, the thermographic peak value is stable, but the base value is incremented. The natural response would be a decrease in the width, area and volume, but that did not happen. On the contrary, those values increased until the welding voltage returned to 19 V and then started decreasing. This behaviour indicates that width, area and volume indeed increase during the voltage step interval, but this change is less noticed since the base value is also increasing.

The relation between the bead width, reinforcement and the voltage is clear too, but the applied stimuli are not disturbing enough. The complex relationship between the welding parameters and the disturbances effect is noted when the weld bead depth changes for no apparent reason, although a small variation of the welding current in position 55 mm is observed.

The autocorrelation and cross-correlation analysis show high values, indicating an adequate sampling time and a no dead time between the bead width and the stimulus.

### 3.2. Modelling and Validation of Estimators

The neural network proposed in Section 2.4 for estimating the depth and width of the weld bead was trained and validated using the experimental data described in Section 3.1. Each experimental dataset was divided in 70% to train, 15% to validate and 15% to test. Experiment 1 was used to model the weld bead depth due to the relationship between this variable and the welding current. In this case, it is modified by the wire feed speed. Experiment 2 was used to model the weld bead width due to the relationship between this variable and the welding voltage, modified in this experiment.

In addition, the complete data set from Experiment 2 was useful for testing the weld bead depth model in different process conditions. Similarly, the complete data set from Experiment 1 was useful for testing the weld bead width model.

The model must represent the dynamic of the welding process. Variations in the measured signal can be induced by measurement noise or by the process dynamics. In the first case, the filter can be useful to reduce the noise in the measurements and improve the modelling. In the second case, the filter eliminates important information of the process and reduces the model accuracy. A mix of both cases can be found in the measurements. In these cases, the use of more complex filters can be necessary. In this work, several training trials were performed using filtered and unfiltered data (inputs and targets). The filter used is the moving average with varying widths between 0–20. This filter is simple to implement in an embedded device. The satisfactory results obtained in each case prove that a more complex filter is not necessary.

Table 3 shows the results of the training and test processes. The quality of the results is measured using the Mean Squared Error (MSE) and the fit metric (R) between the model outputs and the targets.

#### 3.2.1. Training Results

In the weld bead depth modelling, the best result was obtained without filtering. In the weld bead width modelling, the target values were filtered with 10-element window.

The model obtained from the weld bead depth has a fit of 0.99844, a performance (MSE) of 7.6109 × 10^−4^ at epoch 6, a closed-loop performance of 6.4 × 10^−3^ and the network response is satisfactory.

The model of the weld bead width has a fit of 0.9957, a performance (MSE) of 31.008 × 10^−^^4^ at epoch 11, a closed-loop performance of 0.094 and the network response is satisfactory.

**Figure 19:**
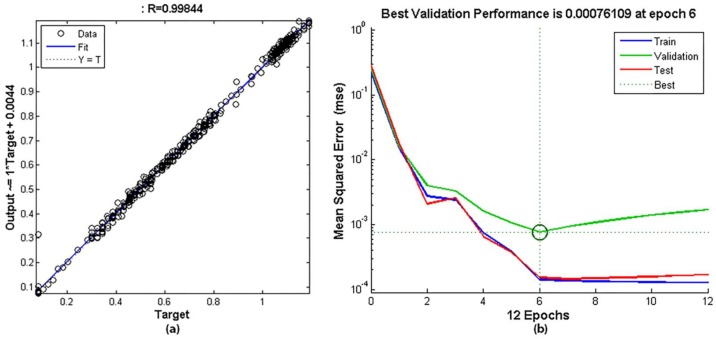
Results in the training of the weld bead depth model: (**a**) Regression curve between the model output and the real depth (target); (**b**) Performance curve in training, validation and test.

**Figure 20:**
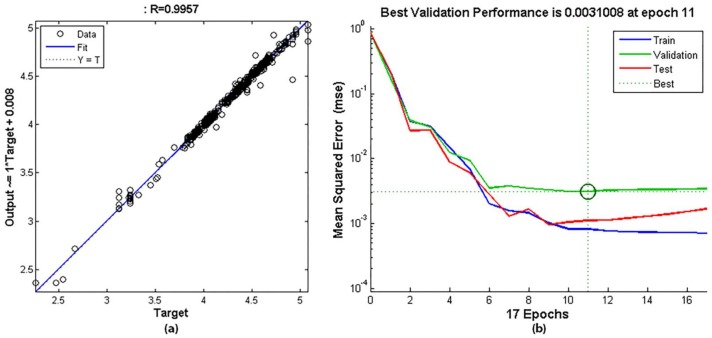
Results in the training of the weld bead width model: (**a**) Regression curve between the model output and the real width (target); (**b**) Performance curve in training, validation and test.

Figures 19a and 20a represent the regression plot between the model outputs and the targets (real weld bead depth or width). The dashed line represents the ideal result (outputs = targets) and the solid line represents the best fit linear regression line. The value of R is an indication of the relationship between the outputs and targets. If R = 1, this indicates that there is an exact linear relationship between outputs and targets. The regression plot for these models achieves an excellent value across the range of measurements.

The performance curves, shown in Figures 19b and 20b do not have indications of overfitting and define the best performance value in epoch 6 and 11 for depth and width respectively. This performance is obtained in open loop and the input $\hat{DW}\left(kT-T\right)$ is substituted by the corresponding target value in the input data set. Performance values show that the estimation errors are very low.

The closed-loop performance is obtained with the feedback activated, such as when the model is used as an estimator in real time. It is natural that it is larger than the previous performance value because the model error is also fed back. For this model, the performance value predicts a good behaviour in a closed loop.

**Figure 21:**
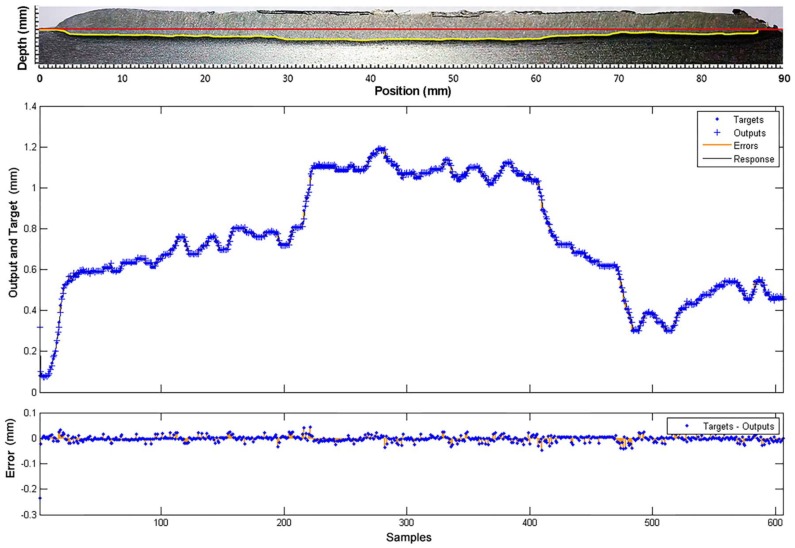
Comparison between the model response (outputs) and the experimental measurements (targets), obtained in the training of the weld bead depth model.

**Figure 22:**
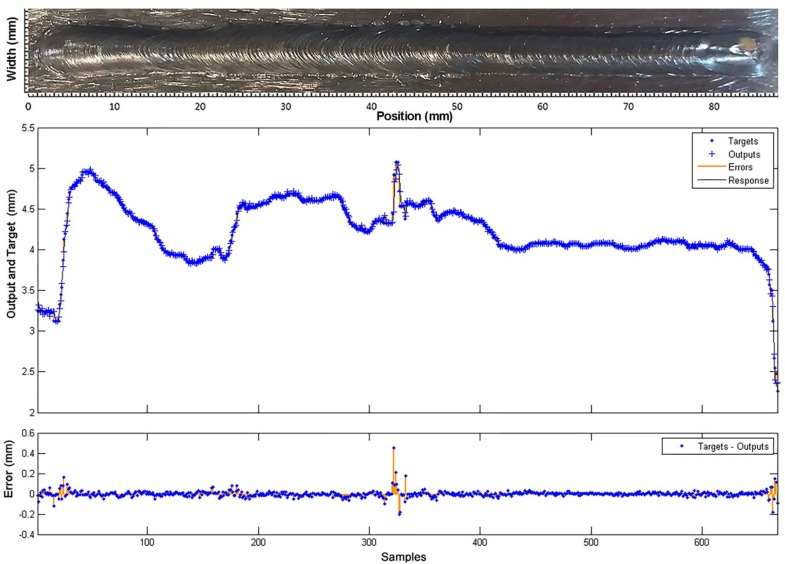
Comparison between the model response (outputs) and the experimental measurements (targets), obtained in the training of the weld bead width model.

In the response curves shown in Figure 21 and Figure 22, the model reproduces the behaviour of the process with high accuracy. The estimation error is very low (less than 0.05 mm for depth and 0.1 for width) all along the curve.

#### 3.2.2. Testing Results

**Figure 23:**
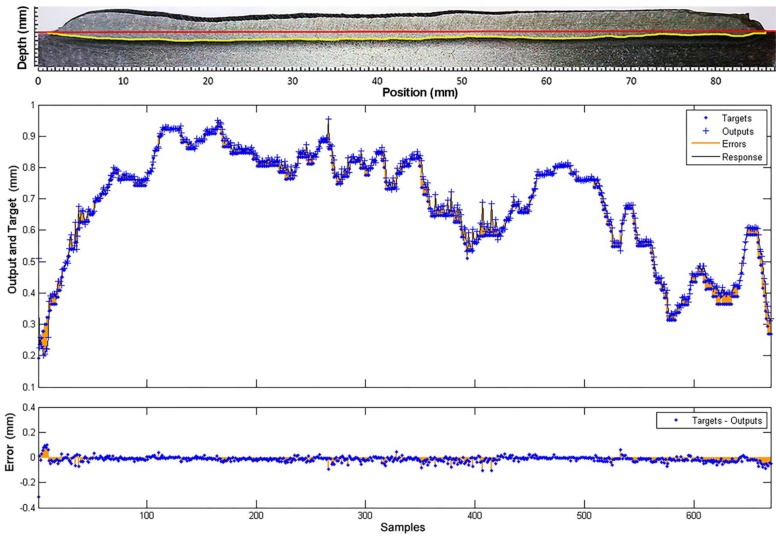
Comparison between the model response (outputs) and the experimental measurements (targets), obtained in the testing of the weld bead depth model with a different data set.

A test of the weld bead depth model, with a different data set, is shown in Figure 23. The dataset was obtained in Experiment 2 with welding voltage variations (see Section 3.1.2). This model has a fit of 0.9901 and a performance of 8.076 × 10^−4^. The closed-loop performance is 0.244 and the estimator response is satisfactory with an estimation error of less than 0.1 mm.

**Figure 24:**
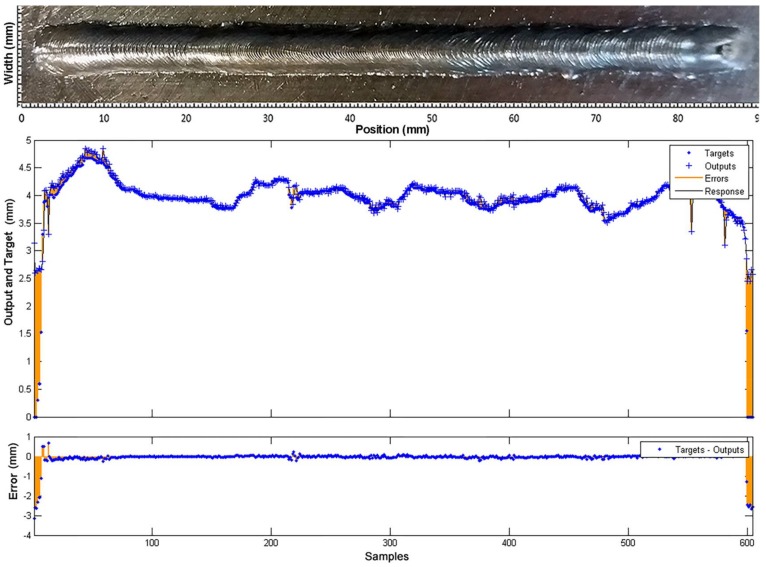
Comparison between the model response (outputs) and the experimental measurements (targets), obtained in the testing of the weld bead width model with a different data set.

The weld bead width model response, with a different data set, is shown in Figure 24. The dataset was obtained in Experiment 1 with welding speed and wire feed speed variations (see Section 3.1.1). The model has a fit of 0.852 and performance is 0.128. The closed-loop performance was 0.67 and the estimator response was acceptable with an estimating error less than 0.3 mm along the curve.

Finally, it is necessary to emphasize that more significant errors are registered at the beginning and the end of the weld bead. These intervals correspond to the opening and closing of the arc, which are very unstable moments of the process and does not respond to the same dynamic, so they do not represent a threat to the quality of the model.

### 3.3. Simulations of Estimators in FPGA Device

The MLP described in Section 2.5 was synthesized using Intel^®^ Quartus^®^ Prime 15.1 (Altera Corp. from San Jose, CA, USA) for the target FPGA (Cyclone V-5CSEMA5F31C6N) kit DE1-SoC from Terasic Inc. (Hsinchu City, Taiwan). The architecture was then simulated using Mentor Graphics QuestaSim 10.6c from Mentor Graphics Corp. (Orlando, FL, USA). Since the floating-point (FP) units have configurable precision, experiments were conducted using FP units with 32, 27, 24 and 18 bits, all using an exponent width of 8 bits and variable sizes for the mantissa.

**Figure 25:**
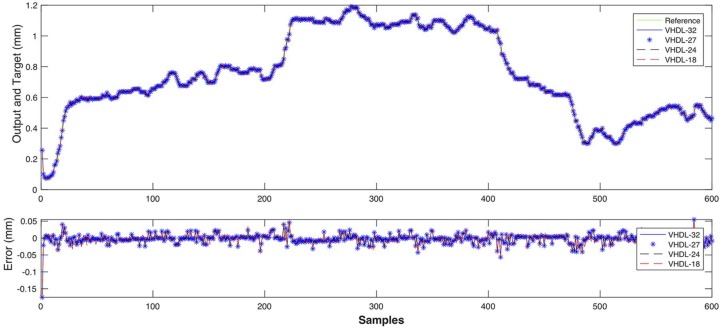
Comparison between the weld bead depth experimental measurements and hardware MPL implementation with various floating-point precisions.

**Figure 26:**
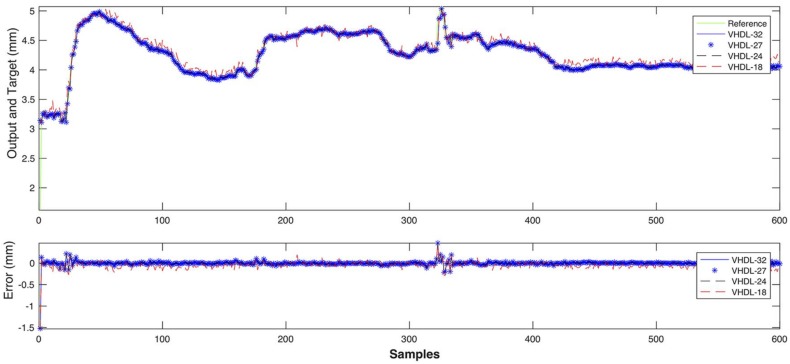
Comparison between the weld bead width experimental measurements and hardware MPL implementation with various floating-point precisions.

Simulation results for the weld bead depth estimator are shown in Figure 25, while results for the weld bead width estimator are presented in Figure 26. In both cases, the simulation output is compared to experimental data measurements and show satisfactory performance for all architectures but the one using 18-bits floating point precision as can be noticed by the noisy red dotted signal in Figure 26.

A more objective error measurement using MSE is presented in Table 4 and confirms the lower accuracy of the 18 bits architecture for the weld bead width estimator.

Finally, FPGA synthesis results are presented in Table 5, where it can be observed that lower bit widths lead to less resource usage as well as faster performance. The chosen architecture was the one using 24 bits of precision which can run at 130 MHz and compute the MLP output in 1.54 us.

### 3.4. The Response of Autoregressive with Exogenous Inputs Linear Models

To compare our results with others methods, two autoregressive with exogenous inputs (ARX) linear models were developed using Matlab’s System Identification Toolbox to estimate the weld bead geometry and compare its performance with neural network models.

The inputs of the models are the same as in the neural network (*T_p_, T_b_, T_a_, T_v_, T_w_, i* or *u*), but removing the delayed variables. The Multi-Input and Single-Output (MISO) model structure and the results of the identification process are shown in Table 6.

**Figure 27:**
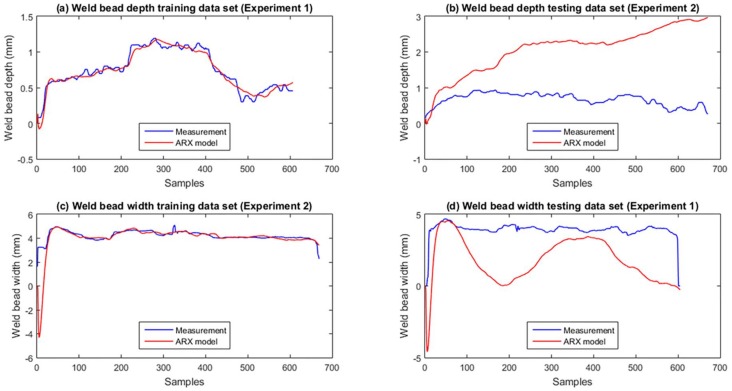
Comparison between the experimental measurements and the response of model ARX.

Similar to the procedure used for the neural network, the measurements obtained in Experiments 1 and 2 were used to identify and test the ARX models. The measurements and model responses are compared in Figure 27. The data set from Experiment 1 was used to identify the depth model (see Figure 27a) and test the width model (see Figure 27d). The data set from Experiment 2 was used to identify the width model (see Figure 27c) and test the depth model (see Figure 27b).

The performance of the ARX models is not satisfactory as shown in the Fit and MSE parameters in Table 6 and the plotted curves in Figure 27. The response of the models with the training data is better than the response with the testing data, as show the MSE values in Table 6 and Figure 27b,d. In all cases, the models showed a very low accuracy compared to the neural network. Tests show that these linear ARX models cannot satisfactorily represent the dynamics of the welding process with the selected inputs and structure.

## 4. Conclusions

The data acquisition and control systems development can be a complex task under arc welding conditions. Related works show the complexity of the arc welding process and the evolution of measurement techniques towards non-contact measurements and intelligent artificial algorithms. This study also shows an open field in the research on the measuring of the weld bead geometry in the electric arc welding process and specifically in the GMAW process.

Sensory fusion techniques have been developed for the estimation of depth and width of the weld bead using a new approach for data processing.

The estimators, based on the fusion of thermographic information and arc welding variables, were implemented using dynamic models and tested with satisfactory results. A novel method to obtain the thermographic features was developed using uncomplex calculations.

The models of depth and width of the weld bead are valid only in the working conditions of the identification experiments. In case of a change in operating conditions, the model must be updated.

A low-cost data acquisition and open-loop control system were developed based on a microcontroller device. It allows sending stimuli to the GMAW process to cause changes in the variables of interest in real-time and captures the dynamics of their behaviour.

An image processing algorithm was developed to measure the depth of the weld bead over its entire length and that guarantees the data to obtain the dynamic model of this magnitude.

A real-time FPGA implementation of the estimators based on an MLP neural network is shown along with a novel method to calculate the weights and bias of the neural network, maintaining the same structure and performance. The architecture used floating-point arithmetic and was experimentally optimized to reduce FPGA resource usage by varying the floating-point bit width. Experimental results obtained by the two dynamic models on the FPGA show high precision and adequate performance being able to compute each estimation in 1.54 us.

The work offers a non-contact measuring tool for researchers and students and can be used in the industry by operators, technicians and unskilled labour.

## Figures and Tables

**Table 1:** Stimulus sequence applied during the experiment with steps in the welding speed and wire feed speed.

Position (mm)	Welding Speed (mm/s)	Welding Voltage (V)	Wire Feed Speed (m/min)	Arc Status
5.0	6.0	20.0	3.0	On
30.0	8.0	20.0	4.0	On
60.0	6.0	20.0	3.0	On
85.0	6.0	20.0	3.0	Off

**Table 2:** Stimulus sequence applied during the experiment with steps in the welding voltage.

Position (mm)	Welding Speed (mm/s)	Welding Voltage (V)	Wire Feed Speed (m/min)	Arc Status
5.0	6.0	19.0	3.5	On
30.0	6.0	21.0	3.5	On
60.0	6.0	19.0	3.5	On
85.0	6.0	19.0	3.5	Off

**Table 3:** Training and test results of the estimators.

Estimator of	Weld Bead Depth		Weld Bead Width	
	Training (Exp. 1)	Test (Exp. 2)	Training (Exp. 2)	Test (Exp. 1)
Fit (R)	0.9984	0.9901	0.9957	0.8516
Epoch	6	-	11	-
Open loop MSE	7.6109 × 10^−4^	8.076 × 10^−4^	31.008 × 10^−4^	0.128
Closed loop MSE	6.4 × 10^−3^	0.224	0.094	0.67

**Table 4:** Comparison between the accuracy of implementations with different floating-point precisions.

MPL Floating Point Precision	Weld Bead Depth Estimator MSE	Weld Bead width Estimator MSE
32 bits	2.095 × 10^−4^	5.045 × 10^−3^
27 bits	2.095 × 10^−4^	5.046 × 10^−3^
24 bits	2.094 × 10^−4^	5.056 × 10^−3^
18 bits	2.460 × 10^−4^	1.186 × 10^−2^

**Table 5:** Summary of resources and performance of implementations with different floating-point precisions.

Architecture Floating-Point Precision	ALMs Usage (Elements/%)	DSPs Usage (Elements/%)	Frequency (MHz)	Time (us)
32 bits	19,809/62%	24/21%	117.08	1.71
27 bits	16,615/52%	24/21%	125.77	1.59
24 bits	14,233/44%	24/21%	130.19	1.54
18 bits	10,502/33%	24/21%	135.72	1.47

**Table 6:** Parameters of autoregressive with exogenous inputs (ARX) linear models.

Model Parameter	ARX Weld Bead Depth Model	ARX Weld Bead Width Model
Order of A (na)	4	4
Order of B+1 (nb)	[4 4 4 4 4 8]	[4 4 4 4 4 8]
Input-output delay (nk)	[1 1 1 1 1 1]	[1 1 1 1 1 1]
Fit Percent (Fit)	79.87%	59.09%
Mean squared error (MSE) in training	0.0037	0.8029
Mean squared error (MSE) in testing	2.4464	6.4616
